# scEWE: high-order element-wise weighted ensemble clustering for heterogeneity analysis of single-cell RNA-sequencing data

**DOI:** 10.1093/bib/bbae203

**Published:** 2024-05-02

**Authors:** Yixiang Huang, Hao Jiang, Wai-Ki Ching

**Affiliations:** School of Mathematics, Renmin University of China, No. 59 Zhong guancun Street, 100872, Beijing, China; School of Mathematics, Renmin University of China, No. 59 Zhong guancun Street, 100872, Beijing, China; Department of Mathematics, The University of Hong Kong, Pokfulam Road, Hong Kong

**Keywords:** scRNA-seq data, Element-wise, High-order Similarity, Ensemble Clustering

## Abstract

With the emergence of large amount of single-cell RNA sequencing (scRNA-seq) data, the exploration of computational methods has become critical in revealing biological mechanisms. Clustering is a representative for deciphering cellular heterogeneity embedded in scRNA-seq data. However, due to the diversity of datasets, none of the existing single-cell clustering methods shows overwhelming performance on all datasets. Weighted ensemble methods are proposed to integrate multiple results to improve heterogeneity analysis performance. These methods are usually weighted by considering the reliability of the base clustering results, ignoring the performance difference of the same base clustering on different cells. In this paper, we propose a high-order element-wise weighting strategy based self-representative ensemble learning framework: scEWE. By assigning different base clustering weights to individual cells, we construct and optimize the consensus matrix in a careful and exquisite way. In addition, we extracted the high-order information between cells, which enhanced the ability to represent the similarity relationship between cells. scEWE is experimentally shown to significantly outperform the state-of-the-art methods, which strongly demonstrates the effectiveness of the method and supports the potential applications in complex single-cell data analytical problems.

## INTRODUCTION

Single-cell RNA sequencing (scRNA-seq) technology has rapidly gained attention since the first release in 2009. With the emergence of large amount of scRNA-seq data, the exploration of data analysis methods has become critical in revealing biological mechanisms. Among them, heterogeneity analysis of scRNA-seq data builds the basis for downstream analysis by revealing cellular complexity, including cell type heterogeneity and the transcriptomic signatures.

In recent years, a great number of algorithms have been proposed [1–4] for addressing the problems of single-cell heterogeneity analysis. pcaReduce [5] presents an agglomerative clustering framework by combining principal component analysis (PCA) and k-means methods for inferring cellular hierarchies. [6] applies PCA algorithm and Laplacian transformation for dimension reduction of scRNA-seq data, and then employs cluster-based similarity partitioning algorithm (CSPA) to robustly analyze the heterogeneity embedded in scRNA-seq data. RaceID [7] assumes that different cell types robustly express some specific ‘outlier’ genes, achieving efficient identification of rare cell types in complex cell mixtures. Among the graph-based algorithms, CIDR [8] uses a simple implicit imputation method to mitigate the impact of data loss in single-cell transcriptome data, and then performs clustering based on the first few major coordinates with principal coordinates analysis. Celltree [9] is based on a powerful Bayesian statistical framework that represents cells as a statistical mixture of classes, allowing the capture of subtle evolutionary features along a continuum of soma and handling heterogeneous groups well. SCENIC [10] uses GENIE3 and GRNBoost (Gradient Boosting) to infer co-expression modules between transcription factors and candidate target genes, thereby providing a clearer data structure for downstream clustering. SIMLR [11] combines multiple kernels to learn the stable distance measure most suitable for the data structure to perform spectral clustering. SNN-Cliq as a graph based method [12] proposes a cluster-based clustering algorithm with a similarity measure based on shared nearest neighbors between cells, which can identify cell clusters of different densities and shapes. [2] proposed a hierarchical clustering framework based on new similarity measure using cell–pair correlation, showing robustness in cellular heterogeneity analysis for small scRNA-seq data sets. A kernel non-negative matrix factorization framework was further developed for dealing with relatively large size of scRNA-seq data in [4].

With the rapid development of deep learning, a lot of methods based on neural networks have been studied in recent years. ScDeepcluster [13] uses an autoencoder network to simultaneously learn low-dimensional feature representation and cluster assignment. ScDCC [3] encodes prior knowledge into constraints based on scDeepcluster method and integrated into the clustering process through a new loss function. scGNN [14] proposed a left-truncated mixture Gaussian model to evaluate heterogeneous gene expression patterns and aggregate cell relationships with graph neural networks. scSemiGAN [15] builds a semi-supervised cell type annotation and dimensionality reduction framework based on the generative adversarial network.

Different methods yielded different results even for the same problems, as no existing method outperforms all the other competitors in all scenarios. Hence it is of great challenge to select the optimal method in single-cell heterogeneity analysis. Therefore, the idea of integrated learning offers a compelling alternative. A lot of works have been done based on weighted fusion of single-cell clustering results. SAFE [16] uses hyper-graph partitioning algorithm to integrate multiple clustering methods to build the final consensus partition. SAME [17] developed a mixture model ensemble clustering method to robustly analyze the cellular heterogeneity in scRNA-seq data. sc-GPE [18] calculates the Adjusted Rand Index (ARI) between the base clustering results, assigning greater weight to base clusterings that are more similar to other results. However, the weight strategy in the above ensemble representatives mainly considers base clustering as a whole, while neglects the element-wise contribution inside base-clustering. Besides, most existing methods ignore the high-order connection information between cells which may encode comprehensive structure information. In this paper, we propose a novel element-wise weighted ensemble method: scEWE, which introduces the first-order and second-order similarity in element-wise weight matrix construction and adaptively optimize to construct the final consensus co-association matrix to capture the high-order similarity relationship between cells as well as guarantee a robust reflection on the relationship between base clusterings. Finally, the obtained weighted consensus matrix is incorporated into the spectral clustering framework with low rank representation to indicate the stable heterogeneity result for scRNA-seq data.

## METHODS

**Figure 1:**
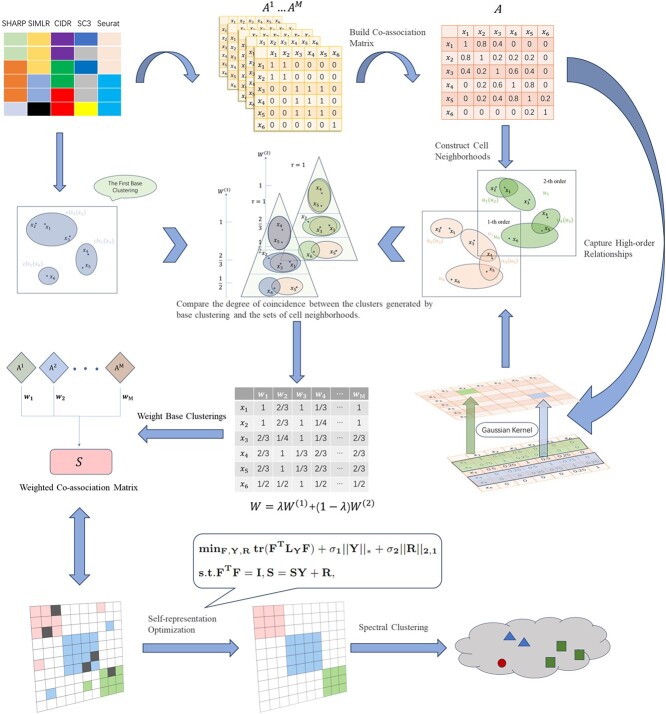
Flowchart for scEWE.

We proposed a high-order Element-wise Weighted Ensemble learning model for single-cell data analysis(scEWE). The architecture of scEWE is shown in Figure 1.

scRNA-seq data usually have tens of thousands of genes in attribute space, with a large number of genes in low expression or undetected, which interferes with the correct identification of cell populations. Based on the characteristics of high-spasity, high-noise and high-dimensionality in scRNA-seq data, we assume a large number of attributes will make little contribution to the clear data representation of the data. On the other hand, highly variable genes are generally considered important for distinguishing cellular heterogeneity. We therefore select the top few highly variable genes with the largest variance to extract the rich information contained in the scRNA-seq data. In data preprocessing stage, we filtered the top 10% of genes with the largest variance.

### Weighted co-association matrix

We let $X = \left \{x_{1}, \cdots , x_{N}\right \}$ be a dataset with $N$ cells, where $x_{i}$ represents the $i$-th cell. We use $P = \left \{\pi ^{1}, \cdots , \pi ^{M}\right \}$ to represent the M base clusterings in the ensemble, where the $M$-th base clustering contains $n^{m}$ clusters. Moreover, $cls_{m}(x_{i})$ denotes the cluster to which the $i$-th cell belongs in the $m$-th clustering.

The co-occurrence matrix $A^{m}$ for $m-$th base clustering is constructed as 


(1) \begin{align*}& A^{m}_{ij} = \left\{ \begin{array}{cc} 1,\quad if \;cls_{m}(x_{i}) = cls_{m}(x_{j})\\ 0,\quad if \; cls_{m}(x_{i}) \neq cls_{m}(x_{j}) \end{array} \right.\end{align*}

Traditional co-association matrix $A$ is constructed by averaging $A^{m}, i=1,2,\ldots M$: 


(2) \begin{align*}& A = \frac{1}{M} \sum_{m = 1}^{M} A^{m}.\end{align*}

One way to improve the representation ability of $A$ is to assign weights $\beta _{m}$ to $A^{m}$: 


(3) \begin{align*} &A = \sum_{m = 1}^{M}\beta_{m} A^{m},\nonumber\\ &\sum_{i=1}^{m}\beta_{m}=1,\beta_{m}\geq 0.\end{align*}

However, due to the diversity of cell clusters, base clusterings that perform well on some cells may perform poorly on other cells. In order to better exploit the strength of base clusterings, we weight the base clusterings at the cell scale.

Specifically, we first construct the initial co-association matrix $A$ according to Eq. (2), and then improve the representation of the co-association matrix by iterative weighting. Given the initial co-association matrix $A$, we construct the $\tau $-nearest neighborhood set of cell $i$ as follows: 


(4) \begin{align*}& u_{i} = \{x_{i_{1}},\cdots,x_{i_{\tau}}\},\end{align*}

where $i_{1},\cdots ,i_{\tau }$ denote the column numbers corresponding to the $\tau $ largest elements in row $i$ of $A$. Note that we base our framework on co-association matrix when constructing the cell neighborhood, which can make full use of global information.

Intuitively, with the increasing of the similarity between the $\tau $-nearest neighbor set of a cell and the cluster to which the cell belongs, the clustered cell becomes more reliable. We use the Jaccard coefficient as a similarity measure between sets, and the weight matrix $W^{(1)}$ is constructed as follows: 


(5) \begin{align*}& W_{ij}^{(1)} =\ Jaccard(u_{i},cls_{j}(x_{i})),\end{align*}

where 


(6) \begin{align*}& Jaccard(Q_{1},Q_{2}) = \frac{|Q_{1}\cap Q_{2}|}{|Q_{1}\cup Q_{2}|}.\end{align*}

Here $Q_{1}$ and $Q_{2}$ represent two arbitrary sets, and $|\cdot |$ denotes the number of elements in the set. The larger $W_{ij}^{(1)}$, the better the clustering effect of the $j$-th base clustering on the $i$-th cell.

To explore multi-scale associations between cells, we construct a second-order co-association matrix to describe the higher order similarity between cells. In real-world scenarios, if two people have many mutual friends, there is a high probability that the two people are acquainted. Inspired by this phenomenon, two cells that are very similar to each other should also belong to the same type. Therefore, the similarity information in the co-association matrix is regarded as a highly condensed sample feature. The Gaussian radial basis function is used to calculate the similarity between cells: 


(7) \begin{align*}& Simil(x_{i},x_{j}) = exp\left(-\frac{||\hat{x_{i}}-\hat{x_{j}}||^{2}}{2\sigma^{2}}\right),\end{align*}

where $\hat{x_{i}}$ represents the $i$-th row data of the initial co-association matrix $A$ (Eq. (2)), $\sigma ^{2} = \frac{1}{N-1}\sum _{i = 1}^{N}||\hat{x_{i}}-\overline{x}||_{2}^{2}$, $\overline{x} = \frac{1}{N}\sum _{i = 1}^{N}\hat{x_{i}}$. Then, we obtain the second-order co-association matrix $A^{(2)}$according to the cell similarity: 


(8) \begin{align*}& A^{(2)}_{ij} = Simil(x_{i},x_{j}).\end{align*}

Denote 


(9) \begin{align*}& u_{i}^{\prime} = \{x_{i_{1}^{\prime}},\cdots,x_{i_{\tau}^{\prime}}\},\end{align*}

where $i_{1}^{\prime},\cdots ,i_{\tau }^{\prime}$ denote the column numbers corresponding to the $\tau $ largest elements in row $i$ of $A^{(2)}$. The second-order weight matrix $W^{(2)}$ is constructed accordingly: 


(10) \begin{align*}& W^{(2)}_{ij} = Jaccard(u_{i}^{\prime},cls_{j}(x_{i})).\end{align*}

We express the final weight matrix $W$ as a linear combination of the first-order and second-order weight matrices: 


(11) \begin{align*}& W = \lambda\cdot W^{(1)} + (1-\lambda)\cdot W^{(2)}.\end{align*}

The parameter $\lambda $ balances the proportion of the first-order and second-order weight matrices. For the convenience of calculation, the matrix $W$ was represented in the form of column vectors $W=\{w_{1},w_{2},\cdots ,w_{M}\}$, where $w_{i}$ is the $i$-th column vector of $W$. The weighted co-association matrix $S$ is computed as 


(12) \begin{align*}& S = \sum_{m = 1}^{M} (w_{m} w_{m}^{T})^{\gamma} \odot A^{m},\end{align*}

where $\odot $ denotes the matrix dot product, and$(\cdot )^{\gamma }$ represents element-wise exponentiation. The scale parameter $\gamma \in [0,\infty )$ measures the importance of the weighting method in the ensemble process. If we want to enhance the influence of cell weights on the weighted co-association matrix $S$, we should set a larger $\gamma $.

We iteratively update the weighting matrix as represented by $W=\{w_{1},w_{2},\cdots ,w_{M}\}$ to ensure a stable $S$. Specifically, for $S_{i}$ we follow the steps of Eqs. (4)–(11) to generate updated weight matrix $\hat{W}=\{\hat{w}_{1},\hat{w}_{2},\cdots ,\hat{w}_{M}\}$ and corresponding $ S_{i+1} = \sum _{m = 1}^{M} (\hat{w}_{m} \hat{w}_{m}^{T})^{\gamma } \odot A^{m} $ until $\mu < 10^{-3}$, where 


(13) \begin{align*}& \mu = \frac{||S_{i+1}-S_{i}||_{F}}{||S_{i}||_{F}}.\end{align*}

### Optimization framework

After obtaining the weighted co-association matrix $S$, a common practice is to use the spectral clustering algorithm to output the clustering results, since $S$ can be viewed as the similarity matrix of the cells: 


(14) \begin{align*} &\text{min}_{F}\:tr(F^{T}L_{S}F) \nonumber\\ &\text{s.t.}\quad F^{T}F=I.\end{align*}

where $L_{S}$ stands for the normalized Laplacian matrix of $S$, and $F$ represents the clustering result matrix.

However, noise in single-cell data often disrupts the data structure, making the clustering results significantly different from the real cell clusters. We regard attribute matrix for each cluster form a subspace of the feature space, and introduce the idea of subspace clustering for low-rank recovery. Here, the cells polluted by noise are considered outliers. In order to make the weighted co-association matrix $S$ have a clearer structure for more accurate clustering results, we iteratively generate a block-diagonal matrix $Y_{N\times N}$ based on robust spectral ensemble clustering [19]. Given $S$, a self-representation $SY$ on a low-rank subspace is used to approximately reconstruct $S$ with reconstruction error $R$. $Y$ is a dictionary coefficient matrix, which is used to extract important features of $S$. It is worth mentioning that the existing theory has proved that when $S$ is used as the dictionary of $S$ itself, the dictionary coefficient matrix $Y$ has excellent properties to facilitate the subsequent clustering. These properties include low rank, block diagonal and approximate symmetry. The optimization function is as follows: 


(15) \begin{align*} &\text{min}_{F,Y,R}\:tr(F^{T}L_{Y}F)+\sigma_{1}||Y||_{*}+\sigma_{2}||R||_{2,1} \nonumber\\ &\text{s.t.}\quad F^{T}F=I,S=SY+R,\end{align*}

where 


(16) \begin{align*}& L_{Y}=I-D_{Y}^{-\frac{1}{2}}((Y+Y^{T})/2+FF^{T})D_{Y}^{-\frac{1}{2}}.\end{align*}

Here, $D_{Y}$ stands for the degree matrix of $(Y+Y^{T})/2+FF^{T}$. The purpose of constructing the Laplacian matrix using $(Y+Y^{T})/2+FF^{T}$ instead of $S$ is to strengthen the block diagonal structure of the cell similarity matrix during optimization.

### Optimization of cluster number *k*

It is of critical significance to give an accurate estimation of cluster number. Inspired by [2], we determined the number of clusters $k$ based on variance analysis. Variance analysis describes the contribution of variation from different sources to the total variation. We regarded the inter-cluster difference after clustering as the inherent difference of different cell clusters (SSW) and regarded the difference between intra-cluster as SSB.

Suppose that cells are clustered into $K$ clusters, where the $k$-th cluster has $n_{k}$ cells. As before, we used $cls(x_{i})$ to indicate the cluster to which the $i$-th cell belongs. Define the $k$-th cluster center as follows:


(17) \begin{align*}& \overline{x}_{k} = \frac{1}{n_{k}}\sum_{i = 1}^{N} x_{i} \cdot 1(cls(x_{i}) = k),\end{align*}

where $1(\cdot )$ is an indicator function. Therefore, accordingly, 


(18) \begin{align*}& \begin{split} SSB& = \sum_{k = 1}^{K} n_{k}(\overline{x}_{k}-\overline{x})^{2},\\ SSW& = \sum_{i = 1}^{N} \sum_{k = 1}^{K} (x_{i}-\overline{x}_{k})^{2}\cdot 1(cls(x_{i}) = k),\\ SST& = \sum_{i = 1}^{N} (x_{i}-\overline{x})^{2}. \end{split}\end{align*}

An effective cluster partition usually results in large inter-cluster distance and small intra-cluster distance. Therefore, we attempt to obtain better cluster partitions by making $Rate=\frac{SSB}{SST}$ as large as possible. However, as the number of cell clusters increases, the Rate value usually increases monotonically. We therefore used the growth rate as a criterion and the optimal cluster number is achieved by solving the following optimization problem: 


(19) \begin{align*}& k^{*} = argmin_{k}\frac{\sum_{i = 1}^{q^{\prime}} Rate_{i}(k+1)-Rate_{i}(k)}{\sum_{i = 1}^{q^{\prime}}Rate_{i}(k)-Rate_{i}(k-1)},\end{align*}

where $Rate_{i}(k)$ denotes the $Rate$ value of the $i$-th feature when the number of clusters is specified as $k$.

The value of $q^{\prime}$ in the above equation represents the number of top differentially expressed genes.

Finally, $F$ learns the cluster partition of cells, and we perform spectral clustering on $FF^{T}$ to get the final clustering result. We summarize the procedure details in Algorithm 1.


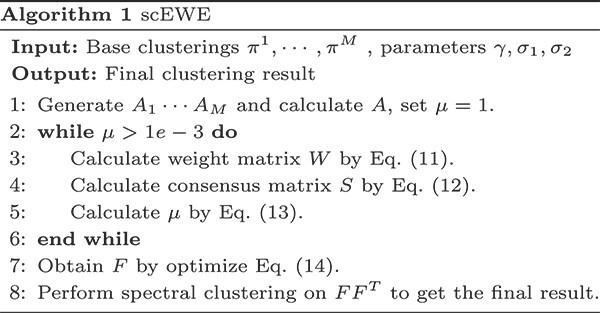


## RESULTS

### Performance evaluation

We use two common indicators, ARI and Nomalized Mutual Information (NMI), to evaluate the performance of scEWE and compared methods. Suppose $P=\{P_{1}\cdots P_{s}\}$ represents the predicted label set and $T=\{T_{1}\cdots T_{r}\}$ represents the real label set, $n_{ij}=|P_{i}\cap T_{j}|$.

Then the ARI is calculated as follows: 


(20) \begin{align*}& ARI(P,T)=\frac{\sum_{ij}\binom{n_{ij}}{2}-[\sum_{i}\binom{a_{i}}{2}\sum_{j}\binom{b_{j}}{2}]/\binom{n}{2}}{\frac{1}{2}[\sum_{i}\binom{a_{i}}{2}+\sum_{j}\binom{b_{j}}{2}]/\binom{n}{2}-[\sum_{i}\binom{a_{i}}{2}\sum_{j}\binom{b_{j}}{2}]/\binom{n}{2}},\end{align*}

where $a_{i}$ is the number of cells labeled $P_{i}$, $b_{j}$ is the number of cells labeled $T_{j}$. The range of ARI is $[-1, 1]$, and a larger value indicates a better clustering result.

NMI uses information entropy theory to describe the similarity of label set distribution, which is calculated as follows: 


(21) \begin{align*}& NMI(P,T)=\frac{\sum_{ij}\frac{n_{ij}}{N}log\frac{Nn_{ij}}{a_{i}b_{j}}}{-\left(\sum_{i}\frac{a_{i}}{N}log\frac{a_{i}}{N}+\sum_{j}\frac{b_{j}}{N}log\frac{b_{j}}{N}\right)/2}.\end{align*}

The range of NMI is $[0, 1]$, and a larger value indicates a better clustering result.

### Compared methods

#### SHARP

As an algorithm for processing large-scale single-cell data, the SHARP method [20] consists of three steps: dividing the cells into different groups, using a random projection algorithm for dimensionality reduction for each group and weighting the results after dimensionality reduction. Finally, SHARP obtains the final clustering result according to the similarity relationship between cells in the consensus matrix.

#### SIMLR

SIMLR [11] uses different distance metrics to construct a nuclear similarity matrix for cells, determines the weight by visualizing each nucleus and then uses spectral clustering to learn cell partitions.

#### CIDR

CIDR [8] uses a simple implicit imputation method to mitigate the impact of dropout in scRNA-seq data, followed by clustering based on the first few principal coordinates.

#### SC3

SC3 [21] uses PCA and Laplacian transformation for dimension reduction processing, and then uses CSPA to partition the consensus matrix. Despite the slow speed when integrating large-scale data sets, SC3 is still one of the mainstream algorithms for single-cell clustering due to its superior performance.

#### Seurat

Seurat [22] first calculates the Euclidean distance of the k nearest cells to each cell in the space after PCA dimensionality reduction, and constructs a k-NN graph. It then refines the edge weights between any two cells based on their shared overlap in their local neighborhood, attempts to partition the graph into highly interconnected communities and finally applies a modular optimization technique (Louvain) to group cells.

#### SAFE

SAFE [16] is an ensemble clustering method for scRNA-seq data. It embeds four state-of-the-art methods, SC3, CIDR, Seurat and t-SNE+k-means for ensemble learning, and used three hypergraph-based partitioning algorithms for final clustering result integration.

#### SAME

SAME [17] is a mixture model-based approach for scRNA-seq data clustering. SC3, CIDR, Seurat, t-SNE + k-means and SIMLR are the five base clustering algorithms. Normalization and transformation of scRNA-seq data are executed in the initial stage. A subset of four diverse sets of clustering solutions are then combined for final cluster ensemble.

### Experiments

We used publicly available datasets from published papers to test the performance of scEWE, and the details of the datasets are shown in Table 1. In data preprocessing stage, we first filtered the top 10% of genes with the largest variance.

**Table 1:** Datasets information

Datasets	Cells	Genes	Clusters
Biase [23]	49	25 737	3
Brain [24]	420	22 085	8
Deng [25]	268	22 431	10
Goolam [26]	124	41 480	5
Treulein [27]	80	23 271	3
Usoskin [28]	622	17 772	4

In the following text, the five single-cell clustering algorithms: SHARP, SIMLR, CIDR, SC3 and Seurat were used to generate base clusterings for our ensemble learning framework. We generated a candidate clustering pool(composed of 50 base clusterings) by constructing 10 base clusterings for each of the five clustering algorithms, through perturbation on the top 10% selected genes with 10 genes taken as intervals, and the parameters are set as $\gamma =0.5,\sigma _{1}=1000,\sigma _{2}=100$ and the maximum number of iterations to 50. Specifically, the 10 base clustering results are generated as follows. Assuming the number of genes in the scRNA-seq dataset is $m$, the base clustering results were generated on the filtered dataset with a number of selected variable genes, where the top variable gene is measured with largest variance in gene expression across all the cells. The number of selected variable genes $n_{g}(i)$ in the $i$-th base clustering result can be represented in the following formula: 


(22) \begin{align*}& n_{g}(i) = 10(i-5)+[m/10], i=1,2,..10,\end{align*}

where $[m/10]$ is the nearest integer around $m/10$. The intuition behind the generation process is as follows. The scRNA-seq data set is sparse and noisy. Hence for each dataset, we use variable gene expression to represent the characteristics of the cells; however, usually different clustering results will be obtained by taking different numbers of variable genes. It is unclear what is the appropriate number of genes that should be involved in the clustering. Therefore, we generate 10 base clustering results for the given dataset with varying numbers of variable genes, which are used for further ensemble learning and may lead to better and more robust results. It is worth mentioning that we take the seed number of 5 for all the random parts in the algorithm, and still take this seed number for comparing methods when comparing the results with the base clustering method.

The parameter $\lambda $ in Eq. (11) plays an important role in balancing the first-order weight matrix and the second-order weight matrix. Motivated by variance analysis, we introduce the measure below to decide an optimal $\lambda $: 


(23) \begin{align*}& \lambda^{*} = \text{argmax}_{\lambda}\frac{SSB(\lambda)}{SST(\lambda)}.\end{align*}

Assume there are $c$ cell sub-populations and each cell belongs to one and only one subpopulation. Treating each cell subpopulation as a treatment group, we can define $SSB(\lambda )$ and $SST(\lambda )$ analogously as Eq. (18). If $c$ cell populations are well separated, $\frac{SSB(\lambda )}{SST(\lambda )}$ is likely to be large. Therefore, the optimal parameter $\lambda ^{*}$ is determined when $\frac{SSB(\lambda )}{SST(\lambda )}$ achieves the maximum.

In order to reduce the computational complexity, when using high-order information, we first filter the base clustering instead of using all the base clustering for ensemble. We preliminarily ran our ensemble algorithm and summed the weight matrix $W$ to evaluate the importance of the base clustering by $ \text{score}_{j}= \sum _{i=1}^{N}W_{ij},j=1\cdots 50. $ The top five base clusterings with the largest score are finally determined and integrated to get the final clustering result. Regarding the parameter $\tau $, the optimal choice of the number of neighbors for each cell is the number of cells in the cluster to which it belongs. We selected larger $\tau $ for the dataset with more genes to better utilize the rich information of cell-to-cell relationships. Therefore, we set the value of $\tau $ according to the following formula $\tau =\lfloor \alpha \frac{N\cdot (2000+f[q-15000])}{k+1}\rfloor ,$ where 


\begin{align*} f(x)= \left\{ \begin{array}{ll} x,& \quad x>0\\ 0,& \quad x\leq 0\\ \end{array} \right.\!. \end{align*}

Here $N$ is the number of cells in the dataset, $q$ is the number of genes and $k$ is the cluster number of cells. $\alpha $ is the data scale parameter, since more cells in large-scale datasets should be selected to construct cell neighborhoods, so we set 


\begin{align*}& \alpha=\left\{\! \begin{array}{ll} \frac{1}{10000},& \quad N<400\\ \frac{1}{3600},& \quad N \geq 400 \end{array} \right.\!. \end{align*}

We compared our method with seven state-of-the-art single-cell clustering methods. Among them, SAFE and SAME are ensemble clustering algorithms developed for single cell. For the five base clustering algorithms, we use the default parameters in both individual setting and ensemble learning. For SAFE and SAME, we also use the default parameters of the methods. It should be noted that cluster number is a critical parameter for the algorithms. In SAFE, SAME and our proposed scEWE where the number of clusters can be determined adaptively, we take the number of clusters determined by the algorithm as input. For the five base clustering methods as comparison methods, we use the real number of clusters for them. Seurat method relies on resolution to determine the number of clusters, and we set the resolution to 1.5. In the SHARP and SC3 method, the seed number is set to 5 uniformly. For the other parameters in Seurat, we specify scale.factor = 10 000, nfeatures=1000, npcs=40 and dims in FindNeighbors=1:10. The results are shown in Tables 2 and 3. SAFE as an ensemble clustering method for scRNA-seq data can demonstrate superiority to SHARP, SIMLR, CIDR and Seurat. In the ensemble comparison partners, SAFE shows better performance in Goolam data, compared with SC3 and SAME. There are no dominant methods that can show best performances in SAFE, SAME and SC3. These methods cannot compete with scEWE in the considered datasets.

**Table 2:** Performance comparison of different methods in ARI on the considered datasets (the best score in each row is highlighted in bold)

	Methods							
Datasets	SHARP	SIMLR	CIDR	SC3	Seurat	SAFE	SAME	scEWE
Biase	$\mathbf{1.0000}$	$\mathbf{1.0000}$	$\mathbf{1.0000}$	$\mathbf{1.0000}$	0.2706	$\mathbf{1.0000}$	$\mathbf{1.0000}$	$\mathbf{1.0000}$
Brain	0.7264	0.5829	0.0815	0.7850	0.5791	0.7580	0.7761	$\mathbf{0.8761}$
Deng	0.3868	0.4125	0.4391	0.6620	0.3565	0.4312	0.4024	$\mathbf{0.6713}$
Goolam	0.6084	0.5441	0.4006	0.5441	0.2330	0.6189	0.4639	$\mathbf{0.8429}$
Treulein	0.1418	-0.077	0.0600	0.4672	0.1501	−	0.1487	$\mathbf{0.5531}$
Usoskin	0.2745	0.6602	0.0289	0.8583	0.4473	0.6377	0.6543	$\mathbf{0.8862}$

**Table 3:** Performance comparison of different methods in NMI on the considered datasets (the best score in each row is highlighted in bold)

	Methods							
Datasets	SHARP	SIMLR	CIDR	SC3	Seurat	SAFE	SAME	scEWE
Biase	$\mathbf{1.0000}$	$\mathbf{1.0000}$	$\mathbf{1.0000}$	$\mathbf{1.0000}$	0.3804	$\mathbf{1.0000}$	$\mathbf{1.0000}$	$\mathbf{1.0000}$
Brain	0.7301	0.7137	0.1748	0.8382	0.6570	0.6804	0.7050	$\mathbf{0.8422}$
Deng	0.4764	0.6625	0.6082	0.8171	0.5475	0.5577	0.5295	$\mathbf{0.8377}$
Goolam	0.5373	0.6637	0.6207	0.6637	0.3447	0.4996	0.5489	$\mathbf{0.7957}$
Treulein	0.1279	0.1318	0.0642	0.5168	0.2653	−	0.0546	$\mathbf{0.6401}$
Usoskin	0.2892	0.7202	0.0567	0.8279	0.5392	0.5820	0.5991	$\mathbf{0.8854}$

In order to compare the embedding capability of the feature space extracted by different methods, we performed t-stochastic neighborhood embedding(tSNE) to visualize the data on a two-dimensional space. For methods that perform clustering based on the consensus matrix (SIMLR, SC3 and scEWE), we use the consensus matrix as input for the tSNE visualization. For methods (CIDR, SHARP and Seurat), we use the embedding matrix obtained for input. Since the SAME and SAFE directly outputs the clustering results, it is hard to evaluate the embedding or modeling capability of the methods and hence incapable for us to show tSNE visualizations for these methods. To visualize the clustering results, we provided UMAP visualizations for all the clustering results by the considered methods. We directly reduce the dimensionality of all datasets by UMAP, and then color the cells according to clustering results of different methods. They are attached in the supplementary file. It can be shown that scEWE helps provide a better clustering result compared with other methods. Besides, we provided the UMAP visualization results of our method and the five base clustering methods for all the datasets in the embedding capabilities and the results were also attached in the supplementary file.

**Figure 2:**
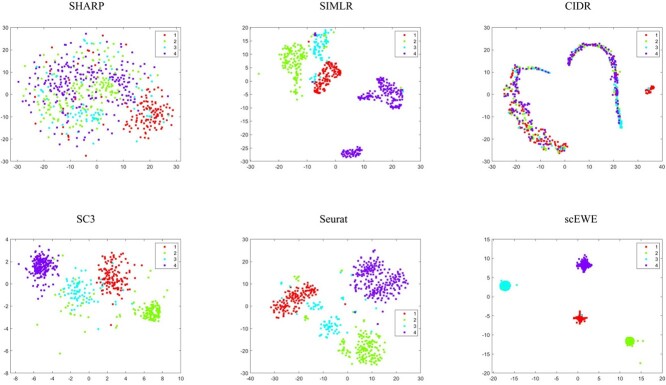
tSNE visualization of embedding capability for the Usoskin dataset. Subfigures correspond to SHARP, SIMLR, CIDR, SC3, Seurat and scEWE. The different colors represent different clusters in true labels.

**Figure 3:**
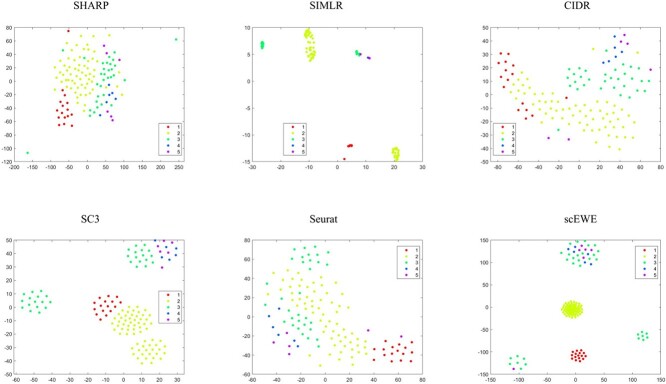
tSNE visualization of embedding capability for the Goolam dataset. Subfigures correspond to SHARP, SIMLR, CIDR, SC3, Seurat and scEWE. The different colors represent different clusters in true labels.

**Figure 4:**
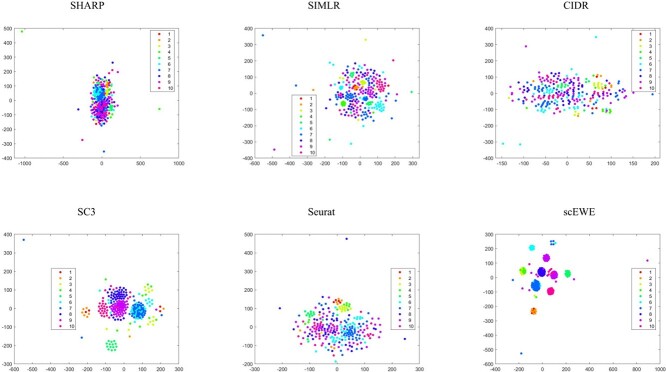
tSNE visualization of embedding capability for the Deng dataset. Subfigures correspond to SHARP, SIMLR, CIDR, SC3, Seurat and scEWE. The different colors represent different clusters in true labels.

**Figure 5:**
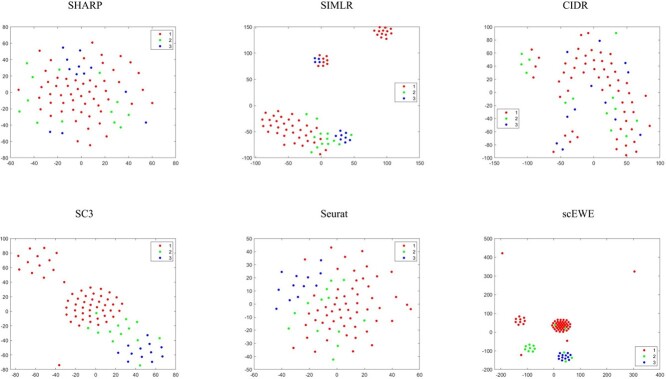
tSNE visualization of embedding capability for the Treulein dataset. Subfigures correspond to SHARP, SIMLR, CIDR, SC3, Seurat and scEWE. The different colors represent different clusters in true labels.

The tSNE visualizations for all the six datasets are included in the supplementary file, where four representative datasets (see Figures 2 to 5) are shown for illustration purpose.

In the Usoskin dataset (Figure 2), CIDR does not capture cellular heterogeneity where different cell types are mixed together. For SHARP, cells of the same type are tightly scattered. Although SIMLR clearly separates cells in different clusters, certain type of cells are divided into two distinct clusters. SC3, Seurat and scEWE showed relatively better descriptions of cellular relationships. In particular, scEWE best captures the relationship between cells, where the same type of cells are tightly clustered and different cell types are well separated. Similar results can be revealed in Figures 3 to 5 that scEWE is among the best method for deciphering the heterogeneity in the considered scRNA-seq data sets.

**Figure 6:**
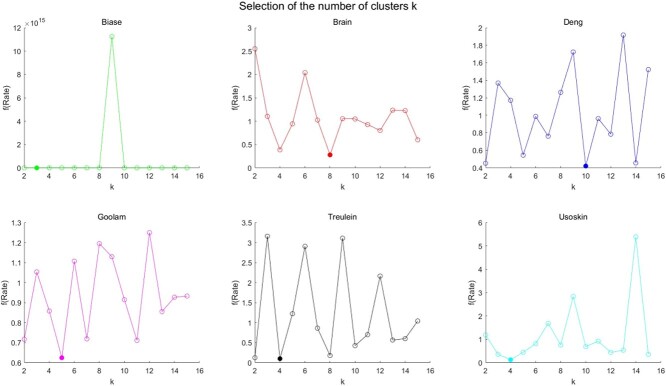
Optimal cluster number determination in different datasets.

We showed the number of clusters predicted by our method in the six datasets, as shown in Figure 6. It is clear that our estimation of the number of cell clusters is relatively robust.

For the single-cell heterogeneity analysis, we used five methods (SHARP, SIMLR, CIDR, SC3 and Seurat) as base clustering, and got an ensemble clustering result (scEWE) with excellent performance. In addition, we proposed a method for estimating the number of cell clusters based on the variance analysis. Experiments have proved that our estimation is very consistent with the real number of cell clusters under the premise that the number of clusters in the base clusterings is accurate.

**Figure 7:**
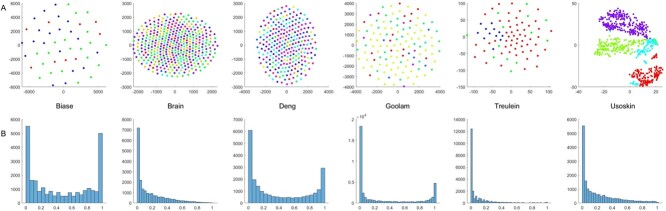
Data distributions in the considered data sets. The upper figures correspond to the tSNE plots in the original data sets; the lower figures correspond to the nonzero ratio distribution of attributes in the data sets.

Some widely used base clustering methods such as CIDR showed unsatisfactory performance on some datasets, hence we tried to analyze the special properties in the considered data sets. We performed tSNE to visualize the original scRNA-seq data to have a understanding on the data distributions, shown in Figure 7(A). It can be seen that most of the data sets were quite noisy where different cell types mixed together, bringing difficulties in correct distinction of cellular heterogeneity. We also evaluated the sparsity of the data by computing the nonzero ratio in each attribute, and see that a number of the attributes are sparsely distributed, shown in Figure 7(B). In particular, the attribute sparsity was clearly shown in Brain, Treulein and Usoskin data. We found that CIDR showed poor performance in these datasets, the possible explanations might be that CIDR was quite sensitive to the sparsity of the data. SHARP, SIMLR and Seurat were less sensitive to the data sparsity compared with CIDR; however, we can see that in Treulein data when almost all the attributes were sparsely distributed, these methods failed to capture the inherent relationship in the data.

## DISCUSSION

### Parameter sensitivity analysis

**Figure 8:**
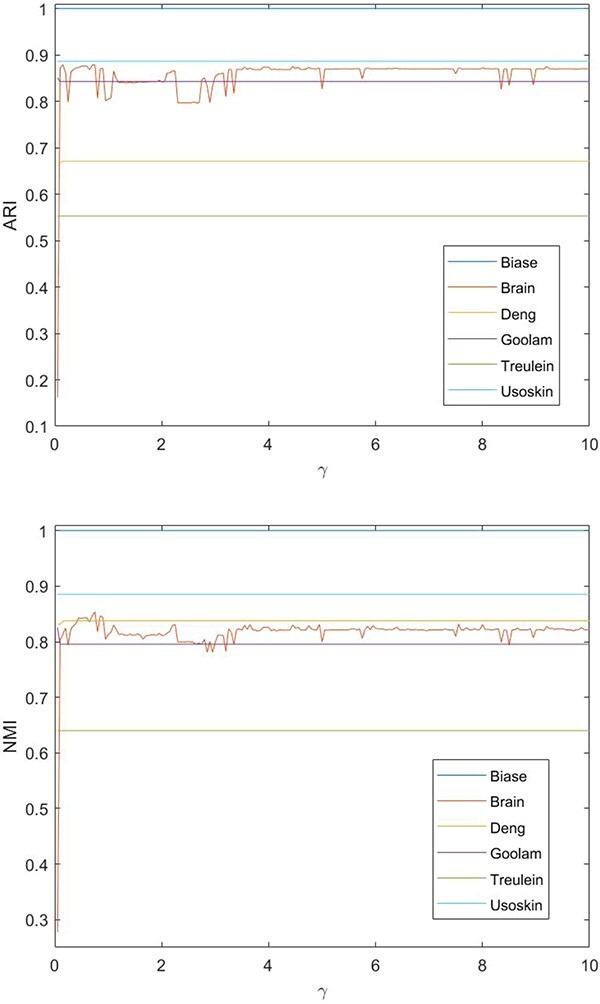
Performance evaluation of scEWE with varied $\gamma $($\sigma _{1}=1000$ and $\sigma _{2}=100$).

**Figure 9:**
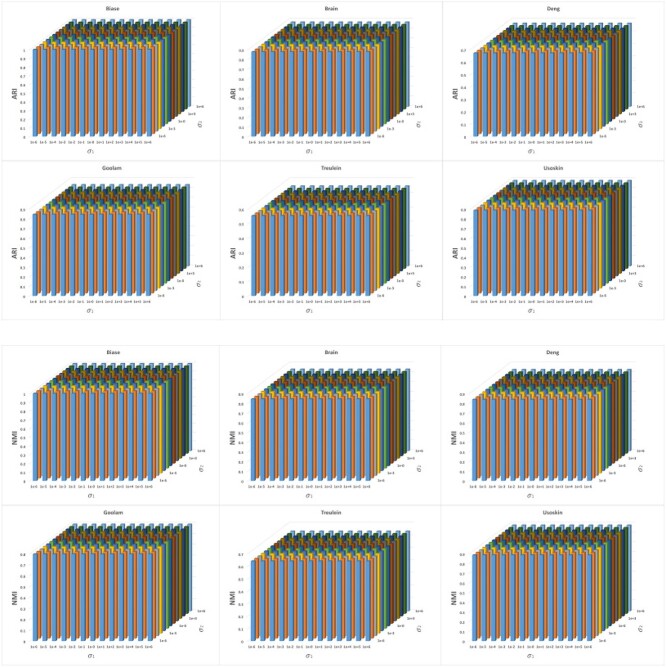
Performance evaluation of scEWE with varied $\sigma $($\gamma =0.5$).

Aiming at investigating the impact of parameters on the performances of the proposed algorithms, we performed a sensitivity analysis on the parameters $\gamma $, $\sigma _{1}$ and $\sigma _{2}$. $\gamma $ is the weighting scale parameter in the element-wise weighting module, which lies in the range $[0, +\infty )$. As $\gamma $ approaches 0, the cell weights play a negligible role in the construction of the co-association matrix, and vice versa. In fact, by adjusting $\gamma $, we can ensure a proper influence on the co-association matrix. $\sigma _{1}$ and $\sigma _{2}$ are the parameters in the optimization module, which represent the contribution of the regularization term in each iteration. In the experiments, the scale parameters $\gamma \in [0,10]$, $\sigma _{1} \in [10^{-6},10^{6}]$ and $\sigma _{2}\in [10^{-6},10^{6}]$ are tested. We show the performance of the model in Figures 8 and 9 as these parameters vary. It is observed that our algorithm exhibits sufficient robustness to the parameter selection. For applications, the parameters $\gamma $ between $[0.5, 1]$ and $\sigma _{i}$ ($i=1,2$) between $[10^{-3}, 10^{3}]$ are preferable as tested to ensure stability and acceptable performance on all the involved datasets.

### High-order information in scEWE

To check the influence of high-order information, we conducted ablation study to our proposed model. We compared the performance of scEWE with and without high-order information in weight matrix construction. In scEWE with high-order information, we first adaptively determine the optimal $\lambda \in (0,1)$, and then use the integrated weight matrix for ensemble learning. The results are shown in the table below. It can be seen that high-order information contribute to the performance in a positive manner.

**Figure 10:**
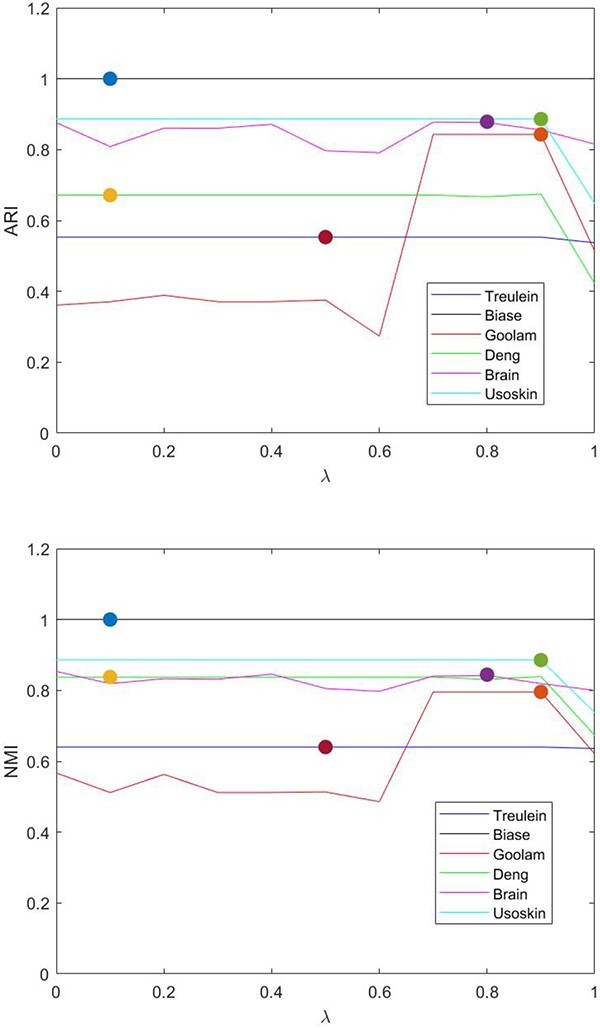
Performance Evaluation of scEWE with varied $\lambda $.

In addition, we check the performance of the model when the value of $\lambda $ varies. The results are shown in Figure 10, where the adaptively selected optimal $\lambda $ for each data set is marked by a bullet. In Figure 10, $\lambda =1$ and $\lambda =0$ indicate the cases where only first-order information and only second-order information is used, respectively. It is shown that the determined $\lambda $ can help achieve optimal or near-optimal performance on the considered datasets. For Deng, Goolam and Usoskin datasets, the model using only first-order information ($\lambda =1$) performs a significant reduction compared with those cases using high-order information ($\lambda <1$). These observations are consistent with the results shown in Table 4 that integration of second-order information would make positive contribution to the model performance. Besides, more observation can be indicated from Figure 10. Particularly, if only second-order information is used (corresponding to $\lambda =0$), the performance of the model is better than that using only first-order information for most datasets. Possible reasons might be that using high-order information can generally capture deep geometric connections between cells. However, an exception is observed for the Goolam dataset, where the clustering performance of the case using only second-order information is inferior to that of other cases where lower order information is used. Therefore, it is beneficial to integrate the first-order and second-order information in a weight-adaptive manner. This observation confirms the reasonability of our model.

**Table 4:** Performance comparison of scEWE with and without high-order information

Datasets	scEWE-with	scEWE-without
Biase	1/1	1/1
Brain	0.8761/0.8422	0.8154/0.7999
Deng	0.6713/0.8377	0.4219/0.6729
Goolam	0.8429/0.7957	0.5128/0.6205
Treulein	0.5531/0.6401	0.5370/0.6361
Usoskin	0.8862/0.8854	0.6474/0.7371

(./.) represents the ARI and NMI value, respectively

### Computational complexity

In order to check the practical applicability of scEWE in diverse scenarios, we conducted analysis detailing the runtime and memory demands. This will help provide a guidance to choose the appropriate base clustering methods for ensemble learning in the real application. We recorded the computational time and memory required to run the base clustering algorithms; the algorithms were run on Windows 10 system with 128GB memory, Inter(R) Xeon(R) Gold 5218 CPU 2.30GHz, and the results were reported in the Tables 5 and 6.

**Table 5:** Runtime comparison on the considered datasets

	Methods					
Datasets	SHARP	SIMLR	CIDR	SC3	Seurat	scEWE
Biase	5.9883 s	11.9106 s	2.1936 s	36.3095 s	2.8102 s	1.6778 s
Brain	15.7061 s	1.6748 m	12.8761 s	1.0250 m	3.1865 s	1.3238 m
Deng	21.4311 s	43.9545 s	7.8914 s	43.2246 s	2.9762 s	25.4687 s
Goolam	9.0592 s	21.1300 s	4.5119 s	36.8681 s	3.9533 s	6.9423 s
Treulein	5.6504 s	15.0118 s	1.6802 s	39.6056 s	1.1788 s	2.7709 s
Usoskin	16.5261 s	3.7770 m	16.0483 s	1.3412 m	2.8122 s	3.3159 m

s represents second, m represents minute

**Table 6:** Memory (MB) requirement comparison on the considered datasets

	Methods					
Datasets	SHARP	SIMLR	CIDR	SC3	Seurat	scEWE
Biase	11.1984	11.1994	37.9880	38.5894	31.4230	9.6484
Brain	72.1460	72.1469	250.9865	222.8073	121.0729	82.1680
Deng	47.2535	47.2544	161.1996	146.9821	109.9423	45.9570
Goolam	41.7846	41.7855	113.9804	133.5275	89.6461	39.3242
Treulein	15.6319	15.6327	37.5795	51.4776	26.3005	14.2578
Usoskin	85.4646	85.4656	304.6056	264.3179	151.3077	84.5781

We now analyze the time complexity in the ensemble learning. Assume that the number of cells is $n$ and the number of base clustering methods is $m$. The co-association matrix initialization takes $O(n^{2})$ time. High-order information extraction involving Gaussian kernel construction has a time complexity of $O(n^{2})$. The time complexity of generating a weighted co-association matrix by weight matrix is $O(mn^{2})$. Assuming that the process takes $t_{1}$ iterations, the time complexity in the weighted co-association matrix construction can be represented as $O([t_{1}(m+1)]n^{2}+2t_{1}\tau mn)$. Assuming spectral ensemble clustering algorithm takes $t_{2}$ iterations to converge, the time complexity of the ensemble learning part can be roughly represented as $O (t_{2}n^{3})$. The computational time and memory requirement for scEWE were shown in Tables 5 and 6. From a computational efficiency perspective, scEWE is efficient when the number of cells in the scRNA-seq dataset is small. However, when the number of cells is relatively large, scEWE shows limited improvement as the ensemble learning sacrifices the computational time to ensure better heterogeneity analysis.

### Cluster number in scEWE

Cluster number is a critical parameter which may influence the model performance. When the estimated number of cell clusters deviates from the ground truth, we conducted experiments to check how the ensemble clustering results will be affected. We varied the number of clusters from 2 to 15 to report the model performance. The results are presented in Tables 7 and 8. We use bold font to indicate the ARI and NMI values corresponding to scEWE with predicted cluster numbers, and underlines to indicate the results with real cluster number. Through the experiments, we have the following interesting discoveries. First of all, scEWE is relatively robust to the number variations in the three datasets Brain, Deng and Goolam. In Brain data, the real cluster number is 8. We can see from Tables 7 and 8 that the performance of scEWE is stable when cluster number is at the neighborhood of 8. In Goolam dataset, similarly, the performance of scEWE is stable when cluster number is at the neighborhood of the real cluster number 5. When the estimated number of cell clusters deviates from the ground truth, we can still get good clustering results with our predicted cluster number. For the other three datasets, the model performance is sensitive to the cluster number. It is therefore of critical significance to give a good estimation on the cluster number. When we compare the performance of scEWE with predicted cluster number to scEWE with real cluster number, we have the following findings. Our proposed variance-analysis-based cluster number estimation method can well capture the distribution characteristics of the dataset. For the Treulein dataset, the true number of clusters is three, while the cluster number estimated by our prediction method is four. The ARI and NMI values of scEWE corresponding to the predicted cluster number though are not globally optimal; they are higher than the corresponding performances under the true number of clusters. For Biase data and Usoskin data, where the real cluster numbers are three and four, respectively, our method can provide accurate cluster number estimations. The performances of scEWE with the predicted cluster number are also the best among the other candidate cluster numbers. These findings suggest the effectiveness of our cluster number method.

**Table 7:** Performance of scEWE in ARI with varied cluster number

	Cluster number													
Datasets	2	3	4	5	6	7	8	9	10	11	12	13	14	15
Biase	0.70	$\mathbf{\underline{1.00}}$	0.68	0.61	0.49	0.27	0.36	0.34	0.27	0.30	0.25	0.17	0.21	0.18
Brain	0.03	0.34	0.59	0.37	0.57	0.78	$\mathbf{\underline{0.88}}$	0.85	0.85	0.57	0.64	0.48	0.55	0.55
Deng	0.10	0.26	0.31	0.37	0.38	0.38	0.39	0.58	$\mathbf{\underline{0.67}}$	0.64	0.33	0.33	0.33	0.33
Goolam	0.46	0.85	0.85	$\mathbf{\underline{0.84}}$	0.84	0.35	0.32	0.26	0.29	0.29	0.23	0.22	0.27	0.21
Treulein	0.30	0.50	$\mathbf{0.55}$	0.31	0.18	0.21	0.18	0.17	0.17	0.18	0.16	0.14	0.15	0.14
Usoskin	0.47	0.67	$\mathbf{\underline{0.89}}$	0.69	0.63	0.54	0.45	0.44	0.44	0.43	0.42	0.42	0.42	0.42

**Table 8:** Performance of scEWE in NMI with varied cluster number

	Cluster number													
Datasets	2	3	4	5	6	7	8	9	10	11	12	13	14	15
Biase	0.79	$\mathbf{\underline{1.00}}$	0.77	0.76	0.70	0.49	0.64	0.60	0.55	0.61	0.55	0.42	0.52	0.47
Brain	0.07	0.47	0.62	0.46	0.63	0.79	$\mathbf{\underline{0.84}}$	0.83	0.82	0.70	0.73	0.70	0.70	0.70
Deng	0.22	0.39	0.45	0.52	0.57	0.57	0.60	0.77	$\mathbf{\underline{0.84}}$	0.82	0.62	0.62	0.62	0.63
Goolam	0.41	0.82	0.84	$\mathbf{\underline{0.80}}$	0.79	0.65	0.63	0.60	0.60	0.58	0.57	0.56	0.55	0.53
Treulein	0.34	0.55	$\mathbf{0.64}$	0.54	0.40	0.45	0.43	0.43	0.43	0.45	0.42	0.39	0.42	0.41
Usoskin	0.53	0.66	$\mathbf{\underline{0.89}}$	0.81	0.77	0.69	0.65	0.64	0.64	0.64	0.63	0.63	0.63	0.63

### Generalization ability

It is necessary to have a clear picture on the property of scEWE if it is robust when the base clustering showed bad performances. We therefore analyzed the impact of base clustering on our ensemble model. On the one hand, we tested whether dropping the worst base clustering result can further improve the ensemble performances. On the other hand, we analyzed whether good base clustering would contribute to the better performance of the ensemble model. ‘Good base clustering’ is denoted if 5% of the cells are wrongly labeled, and ‘perfect base clustering’ is denoted if all the cells are accurately labeled. We therefore replaced the worst base clustering respectively with ‘good base clustering’ and ‘perfect base clustering’ in turn, respectively, to check the influence of them to scEWE. The results for the two scenarios of replacement are reported in Tables 9 and 10, respectively. It is interesting to see that scEWE is robust when the base clustering showed bad performance. When we dropped the worst base clustering, the ensemble model remained stable in Biase, Brain, Goolam and Usoskin data. In Deng and Treulein data however, the ensemble model showed degraded performance. Possible explanations might be that the worst base clustering still contains useful information where the ensemble model scEWE can extract. On the other hand, when we replaced the worst base clustering with ‘good base clustering’ and ‘perfect base clustering’, respectively, scEWE can benefit from the clustering results and showed improved performances.

**Table 9:** Impact of base clustering to scEWE in ARI

	Methods			
Datasets	Drop worst	scEWE	Replace worst as good	Replace worst as perfect
Biase	1.0000	1.0000	1.0000	1.0000
Brain	0.8761	0.8761	0.8761	0.8761
Deng	0.6713	0.6713	0.6713	0.6747
Goolam	0.8429	0.8429	0.9485	1.0000
Treulein	0.5531	0.5531	0.5531	0.5531
Usoskin	0.8827	0.8862	0.9972	1.0000

**Table 10:** Impact of base clustering to scEWE in NMI

	Methods			
Datasets	Drop worst	scEWE	Replace worst as good	Replace worst as perfect
Biase	1.0000	1.0000	1.0000	1.0000
Brain	0.8422	0.8422	0.8422	0.8422
Deng	0.8377	0.8377	0.8377	0.8392
Goolam	0.7956	0.7957	0.8853	1.0000
Treulein	0.6401	0.6401	0.6401	0.6401
Usoskin	0.8785	0.8804	0.9931	1.0000

The rapid advancement of scRNA-seq technologies enables the generation of more and more large-scale datasets, bringing new challenges in computational cost and effectiveness. scEWE as an element-wise weighted ensemble clustering fully considers the neighborhood relationship between cells, and improves the clustering accuracy at the cost of computational complexity. The advantage of scEWE lies in that it is a very flexible framework in adopting different base clustering methods for ensemble learning, and hence can be properly extended for dealing with scRNA-seq data on a relatively large scale. For the large-scale version, scEWE adopted base clusterings with reasonable computational cost, and used first-order information to guarantee efficient element-wise weight matrix learning and hierarchical clustering. For a dataset of more than 1000 cells, we adopted the extended version of the model. In the base clustering generation stage, SIMLR due its inefficiency was removed. The model was tested on the Klein dataset [29] and Baronh dataset [30], which contains 2717 and 8569 cells, respectively, with detailed data information in Table 11 and the performances were shown in Tables 12 and 13. Compared with the state-of-the-art methods, scEWE still demonstrates good performances. Regarding the time complexity, the extended large-scale version of scEWE has a time complexity of $O(n^{3})$. The computational time and memory occupation comparison in large-scale data was shown in the Tables 14 and 15. It can be seen that efficient element-wise weight matrix learning without incorporating high-order information guarantees efficiency and makes the applications to large-scale scRNA-seq data analysis possible. Furthermore, we would like to check whether high-order information incorporation can help improve clustering performance for relatively large-scale data. Table 16 therefore recorded performance of scEWE with and without high-order information for the two considered datasets. Our experiments demonstrated that scEWE incorporating high-order information shows limited better performance for large-scale data with a sacrifice of significant computation burden. Comparing the results for small- and large-scale data, we have the following findings. If the scale of scRNA-seq data is small, the first-order information provides considerably limited understanding for data relationship, and thus, the integration of high-order information is necessary to learn a deep geometric relationship. This reveals the inherent principle of the proposed method. However, if the scale of scRNA-seq data is relatively large, incorporating high-order information can only make limited performance improvement because the first-order information can provide sufficient understanding for establishing an appropriate element-wise weighted co-association matrix. Considering the high computation cost and limited performance improvement for the proposed scheme incorporating high-order information, element-wise weighted ensemble clustering with first-order information might be preferable for the scenario of large-scale scRNA-seq data.

**Table 11:** Large-scale datasets information

Datasets	Cells	Genes	Clusters
Klein [29]	2717	24 175	4
Baronh [30]	8569	20 125	14

**Table 12:** Performance comparison in Klein dataset for each method

	Methods						
Index	SHARP	CIDR	SC3	Seurat	SAFE	SAME	scEWE
ARI	0.5067	0.1770	0.8669	0.3904	0.7583	0.5485	0.8587
NMI	0.5152	0.2790	0.8302	0.4928	0.7419	0.6057	0.8890

**Table 13:** Performance comparison in Baronh dataset for each method

	Methods						
Index	SHARP	CIDR	SC3	Seurat	SAFE	SAME	scEWE
ARI	0.7844	0.3574	0.5708	0.4046	−	0.5942	0.9044
NMI	0.6788	0.5092	0.7021	0.5850	−	0.7037	0.8508

**Table 14:** Runtime comparison for large-scale datasets

	Methods					
Datasets	SHARP	SIMLR	CIDR	SC3	Seurat	scEWE
Klein	1.1547 m	−	1.8740 m	7.4862 m	12.0647 s	12.4229 s
Baronh	1.2820 m	−	29.9062 m	1.9363 h	15.3685 s	1.5925 m

s represents second, m represents minute, h represents hour

**Table 15:** Memory (MB) requirement comparison on large-scale datasets

	Methods					
Datasets	SHARP	SIMLR	CIDR	SC3	Seurat	scEWE
Klein	502.7797	−	1843.2014	1593.7275	1049.7111	502.1133
Baronh	659.6358	−	3854.8459	2911.0148	1117.0372	1318.2852

**Table 16:** Performance comparison of scEWE in large datasets with and without high-order information

Datasets	scEWE-with	scEWE-without
Klein	0.8592/0.8900	0.8587/0.8890
Baronh	0.9053/0.8545	0.9044/0.8508

(./.) represents the ARI and NMI value, respectively

As an ensemble algorithm, our method has good scalability and generalization ability. Users can specify any of the five clustering methods for integration. For the clustering results generated by other single-cell methods, we can also input them as base clusterings into our ensemble framework. To verify the effectiveness of our algorithm, we applied scEWE to five additional clustering tasks. The five datasets including Glass, IS, MNIST, Texture and Ionosphere (Table 17) are publicly available and can be obtained from [31]. Following [31], we constructed a large pool of candidate base clusterings, and each base clustering was generated by the $k$-means algorithm, and the number of clusterings was randomly selected in the range $[2,\sqrt{N}]$, where $N$ is the number of samples.

**Table 17:** Datasets information

Datasets	Samples	Features	Clusters
IS	2310	19	7
Texture	5500	40	11
MNIST	5000	784	10
Ionosphere	351	34	2
Glass	214	9	6

We generated 100 base clusterings for each dataset to form the base clustering pool. In each experiment, 10 base clusterings were chosen in a perfectly equally probable manner. Compared with five methods [31], [32] and [33], we provided the mean performance (NMI and ARI) and standard deviation and marked the best score for each dataset in bold. Similar to the above, we set $\lambda =1,\tau =5,\gamma =0.5$. After sample weighting by our method to get the consensus matrix, we used hierarchical clustering to generate the final clustering results. The results were shown in Tables 18 and 19, and it can be seen that our method exhibits excellent performance in this challenging test.

**Table 18:** Performance evaluated in ARI on extended datasets (the best score in each row is highlighted in bold)

	Methods					
Datasets	LWEA	LWGP	ECPCS-MC	ECPCS-HC	LRTA-EA	scEWE
IS	$0.509\pm 0.036$	$\mathbf{0.536} \pm 0.037$	$0.513\pm 0.049$	$0.504\pm 0.042$	$0.178\pm 0.238$	$0.514\pm 0.022$
Glass	$0.261\pm 0.012$	$0.266\pm 0.005$	$0.262\pm 0.015$	$0.260\pm 0.016$	$0.011\pm 0.020$	$\mathbf{0.272}\pm 0.023$
Ionosphere	$0.144\pm 0.063$	$0.163\pm 0.007$	$0.115\pm 0.060$	$0.095\pm 0.096$	$0.025\pm 0.033$	$\mathbf{0.308}\pm 0.213$
MNIST	$0.539\pm 0.025$	$0.496\pm 0.020$	$0.514\pm 0.046$	$0.483\pm 0.036$	$0.549\pm 0.015$	$\mathbf{0.557}\pm 0.030$
Texture	$0.668\pm 0.033$	$0.632\pm 0.032$	$0.595\pm 0.068$	$0.589\pm 0.046$	$0.664\pm 0.036$	$\mathbf{0.677}\pm 0.062$

**Table 19:** Performance evaluated in NMI on extended datasets (the best score in each row is highlighted in bold)

	Methods					
Datasets	LWEA	LWGP	ECPCS-MC	ECPCS-HC	LRTA-EA	scEWE
IS	$0.616\pm 0.029$	$0.642\pm 0.026$	$0.612\pm 0.028$	$0.598\pm 0.025$	$0.245\pm 0.312$	$\mathbf{0.662}\pm 0.021$
Glass	$0.375\pm 0.019$	$0.383\pm 0.014$	$0.364\pm 0.021$	$0.369\pm 0.035$	$0.410\pm 0.017$	$\mathbf{0.428}\pm 0.033$
Ionosphere	$0.117\pm 0.016$	$0.116\pm 0.006$	$0.081\pm 0.042$	$0.097\pm 0.034$	$0.219\pm 0.034$	$\mathbf{0.243}\pm 0.174$
MNIST	$0.638\pm 0.016$	$0.623\pm 0.019$	$0.624\pm 0.032$	$0.589\pm 0.035$	$0.642\pm 0.017$	$\mathbf{0.668}\pm 0.015$
Texture	$0.764\pm 0.021$	$0.749\pm 0.019$	$0.723\pm 0.038$	$0.717\pm 0.031$	$0.781\pm 0.024$	$\mathbf{0.811}\pm 0.029$

### Comparison with deep learning algorithms

To better demonstrate the superiority of scEWE, we compared scEWE with efficient deep learning methods scDeepCluster and scGNN. Since scDeepCluster and scGNN allow only count data for processing, if the dataset was not count data, necessary preprocessing was conducted to ensure the implementation of the algorithms. Besides, deep clustering methods such as SDCN [34] and DFCN [35], which integrate high-order structural information into clustering processes, were also introduced for method comparison. For scDeepCluster, we set the number of clusters to the actual number of clusters, the number of pre-trained epochs to 700, the maximum number of iterations to 5000 and the remaining parameters to the default values. For scGNN, we use ‘Do not infer LTMG mode’ with default parameters. In the construction of k-nearest neighbor graph, k is set to 100 and the maximum number of iterations of the model is set to 2000. For SDCN, we set the number of input nodes to the number of genes in the dataset, the number of clusters to the number of real classes, the training batches to 800, the k value in the k-nearest neighbor map to 50 and the remaining parameters to their default values. For DFCN, we set the training epoch to 7000, set the number of clusters to the number of real classes, set the number of input nodes to 100 and use the default values for the rest of the parameters. The results were shown in Table 20. scDeepCluster demonstrated better performances among the considered deep learning methods. When the number of cells was relatively small, most of the deep learning methods seemed to perform in an unsatisfactory manner. In Klein or Baronh data set, where the number of cells was 2717 and 8569, respectively, scGNN and scDeepCluster showed good performances in capturing the heterogeneity embedded in the single-cell data. In comparison, SDCN and DFCN cannot compete with scGNN and scDeepCluster that are particularly designed for scRNA-seq data. From the results we can see that scEWE can still demonstrate its superiority and effectiveness in dealing with noisy scRNA-seq data.

**Table 20:** Performance comparison with deep learning methods

	Methods				
Datasets	scGNN	scDeepCluster	SDCN	DFCN	scEWE
Biase	0.8116/0.7915	1.0000/1.0000	0.7151/0.7604	0.8590/0.8705	1.0000/1.0000
Brain	0.5231/0.6266	0.5780/0.7035	0.5128/0.6429	0.4609/0.5687	0.8761/0.8422
Deng	0.3867/0.5821	0.3719/0.5988	0.4227/0.6147	0.1864/0.4343	0.6713/0.8377
Goolam	0.3997/0.5494	0.4411/0.5892	0.6046/0.5193	0.6903/0.7101	0.8429/0.7957
Treulein	0.2913/0.2830	0.0197/0.0338	0.0424/0.1351	0.3440/0.3991	0.5531/0.6401
Usoskin	0.3039/0.3923	0.3808/0.4335	0.6041/0.6146	0.0292/0.1599	0.8862/0.8804
Klein	0.7629/0.7901	0.8084/0.8351	0.6735/0.7021	0.6849/0.6637	0.8587/0.8890
Baronh	0.8219/0.7043	0.8743/0.7842	0.5384/0.5839	0.6323/0.6519	0.9044/0.8508

### Biomarker identification

We further hope to dissect the biomarker identification results provided by scEWE. For the brain data set which contains 420 cells in total, we conducted scEWE to get a heterogeneity analysis result for the cells. We further conducted statistical analysis to compare the differences between specific cluster and the remaining clusters by scEWE, to investigate the potential of scEWE in cellular state identification. We performed t-test of the hypothesis that the two independent samples generated by the specific cluster and the remaining clusters come from distributions with equal means, and returns the result of the test in $H$. Here $H=0$ indicates that the null hypothesis (‘means are equal’) cannot be rejected at the $5\%$ significance level. Here $H=1$ indicates that the null hypothesis can be rejected at the $5\%$ level. The top identified markers for the eight clusters in brain data are as follows: cluster 1:‘ADCY8’, ‘A2M’, ‘ACADL’, ‘AGBL5’, ‘ADIPOR1’; cluster 2:‘ADAMTSL4’, ‘ACTN2’, ‘AADAT’, ‘ADCK2’, ‘ABCA11P’ ; cluster 3: ‘ADIPOQ’, ‘ADH1C’, ‘ACSM1’, ‘ADAM29’, ‘ACTR6’ ; cluster 4: ‘ADRA1B’, ‘ADAM19’, ‘ACAN’, ‘ADRB1’, ‘ADM’ ; cluster 5: ‘A4GALT’, ‘ACLY’, ‘ABCA5’, ‘ABCB8’, ‘ACVRL1’ ; cluster 6: ‘ACADSB’, ‘ACRBP’, ‘ACP5’, ‘ACOT4’, ‘AASDH’; cluster 7: ‘ACTN3’,‘ACTRT1‘; cluster 8:‘ABI3BP’. For example, in cluster 2, the identified biomarkers include ‘ADAMTSL4’, ‘ACTN2’, ‘AADAT’, ‘ADCK2’, ‘ABCA11P’. And we found that ADAMTSL4 as a secreted glycoprotein may become a novel immune-related biomarker for primary glioblastoma multiforme (GBM) [36]. AADAT is the enzyme responsible for the formation of the majority of neuroactive kynurenic acid in the brain [37]. ADCK2 is necessary for cell proliferation of GBM, a fatal primary brain tumor containing countless genetic and epigenetic alterations. These findings further support the capability of scEWE in biomarker identification.

### Understanding biological process

**Figure 11:**
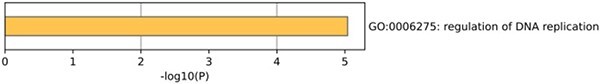
Top biological process item in [38] by Metascape.

To further explore the capability of scEWE in revealing biological process, we investigated on the biological role of the marker genes that scEWE extracted. We performed heterogeneity analysis on the dataset obtained from [38] containing 124 individual cells in various developmental stages from human preimplantation embryos. The major type of cells identified by scEWE was used for further marker gene analysis. The top 10 differentially expressed genes are ‘TERF1’, ‘HESRG’, ‘CD24’, ‘PRDX6’, ‘AASS’, ‘SEPHS1’, ‘NUCKS1’, ‘M6PR’, ‘PHF17’, ‘TUBB’. We performed functional enrichment analysis with Metascape (https://metascape.org). Pathway and process enrichment analysis has been carried out with Gene Ontology Biological Processes. The top extracted biological process is shown in Figure 11.

Take a further look at the marker genes, through Genecards (https:/www.genecards.org/) we found that ‘TERF1’ as Telomeric Repeat-Binding Factor 1 involves in the biological processes of cell cycle, cell division and mitosis. ‘HESRG’ is Embryonic Stem Cell Related Protein, and ‘CD24’ has a pivotal role in cell differentiation of different cell types. These findings suggest the roles of genes involving in cell development such as DNA replication, cell differentiation, etc.

## CONCLUSIONS

In this paper, we have proposed a high-order element-wise weighted ensemble method of heterogeneity analysis for scRNA-seq data: scEWE. Different from traditional weight strategy in ensemble clustering, scEWE novelly incorporates element-wise contribution in each base clustering for weighted co-association matrix construction. A low-rank self-representation framework is incorporated for generating final heterogeneity results. Compared with state-of-the-art methods for scRNA-seq data analysis, scEWE shows robustness as well as superiority in performance. Due to the high complexity of the algorithm, it is worth noting that our method has limited capacity for datasets involving very large number of cells. How to optimize the algorithm to improve the computational efficiency is the work we will consider in the future.

**Key Points**

- We developed a novel ensemble learning framework: scEWE to deal with heterogeneity analysis problem for scRNA-seq data.
- A high-order element-wise weighting strategy was proposed for building the ensemble learning framework.
- Variance-analysis-based low-rank self-representation optimization model was applied for latent embedding and heterogeneity analysis.
- The effectiveness of scEWE was demonstrated through extensive experiments in real-world datasets.

## Supplementary Material

Huangyixiangv1_bbae203

Jianghaov1_bbae203

WKChingv1_bbae203

## Author Biographies

**Yixiang Huang** received the B.Sc. degree from the school of Mathematics and Statistics, Shandong University, China, in 2021. He is a postgraduate student with the School of Mathematics, Renmin University of China, Beijing, China. His research interests include data mining, learning-based modeling and bioinformatics.

**Hao Jiang** received the B.Sc. degree from the Harbin Institute of Technology, Harbin, China, in 2009, and the Ph.D. degree from the University of Hong Kong, Hong Kong, in 2013. She is an Associate Professor with the School of Mathematics, Renmin University of China, Beijing, China. Her research interests include data mining, learning-based modeling, optimization and control of complex systems.

**Wai-Ki Ching** received the BSc degree (Hons.) and the MPhil degree from The University of Hong Kong, Hong Kong, and the PhD degree from the Chinese University of Hong Kong, Hong Kong. He is currently a professor with the Department of Mathematics, The University of Hong Kong, Hong Kong. He is also a visiting fellow with Hughes Hall, Cambridge University, Cambridge, United Kingdom. His current research interests include stochastic modeling, matrix computations, the applications of mathematical models and numerical algorithms in solving problems related to Markov chains, bioinformatics, mathematical finance and management science.
