# Supplementary figures and images for: scEWE: high-order element-wise weighted ensemble clustering for heterogeneity analysis of single-cell RNA-sequencing data
Source: Brief Bioinform. 2024 May 2;25(3):bbae203. doi: 10.1093/bib/bbae203 (PMC11066953; doi:10.1093/bib/bbae203)

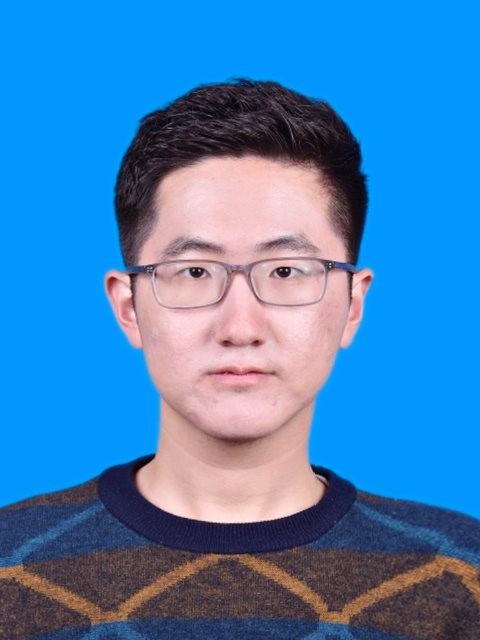

Supplement: Huangyixiangv1_bbae203 [file huangyixiangv1_bbae203.jpeg]

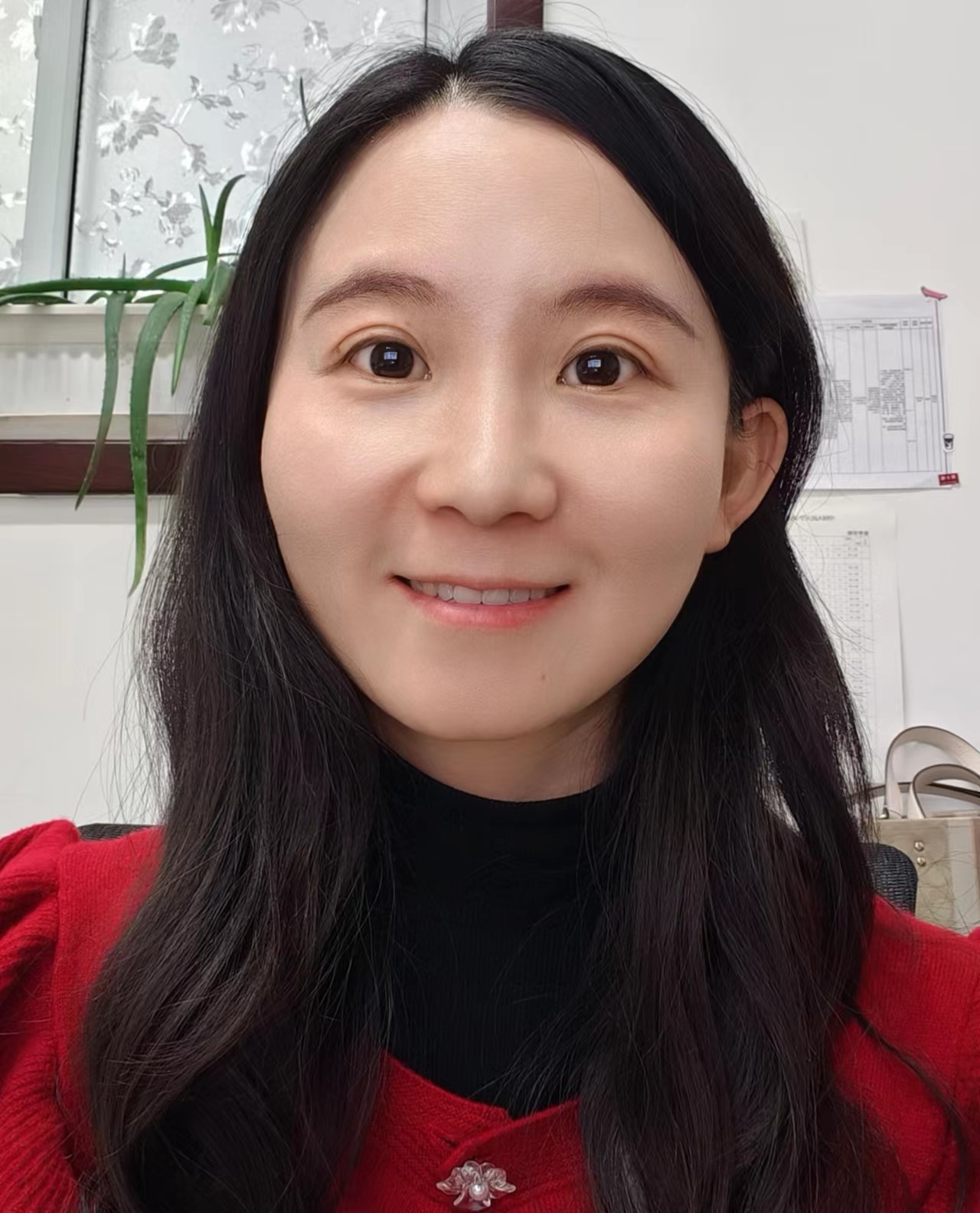

Supplement: Jianghaov1_bbae203 [file jianghaov1_bbae203.jpeg]

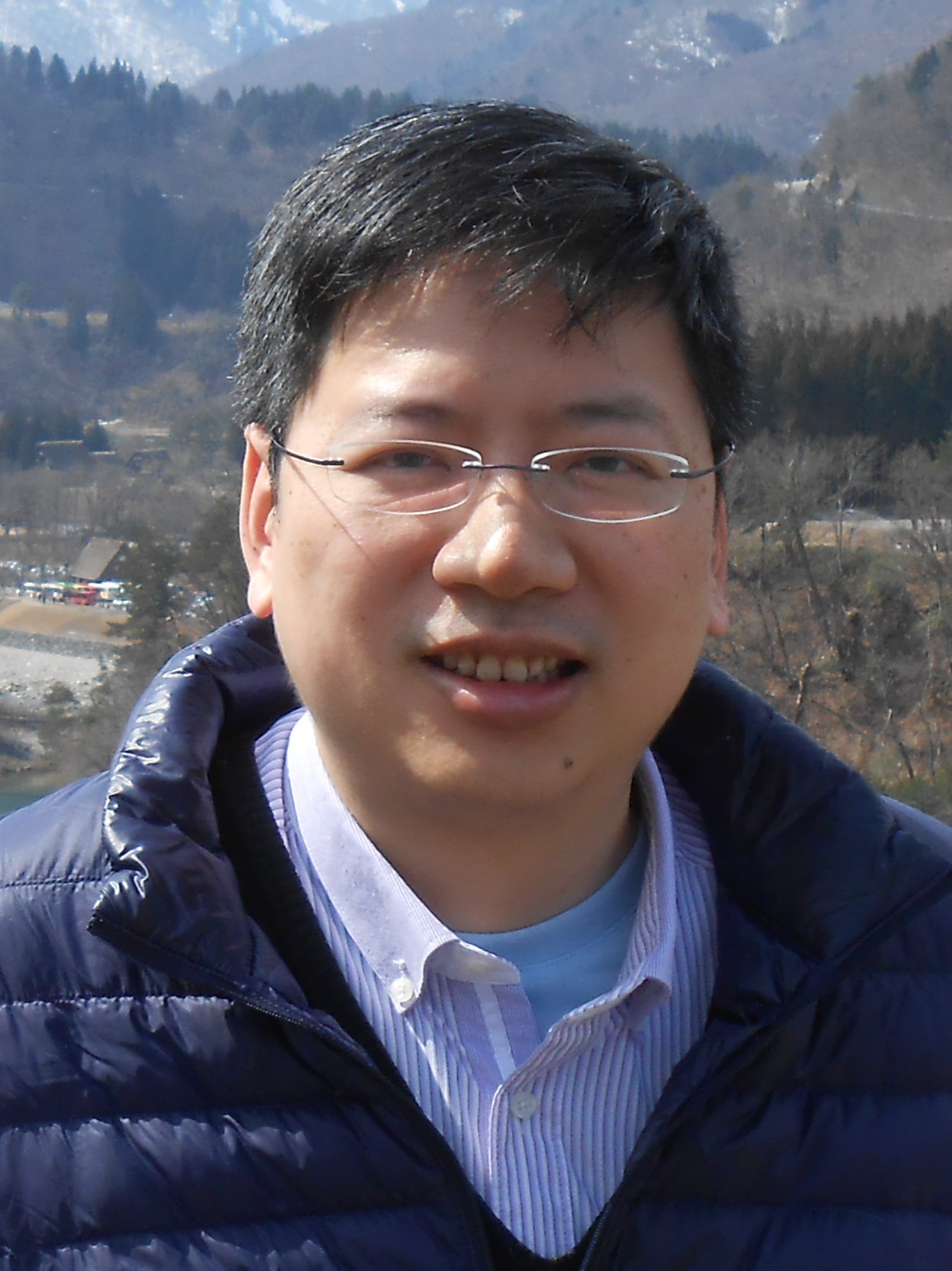

Supplement: WKChingv1_bbae203 [file wkchingv1_bbae203.jpeg]
